# Treatment Strategy of Fractured Osteochondroma in the Young Athlete’s Knee

**DOI:** 10.3390/jcm12113615

**Published:** 2023-05-23

**Authors:** Hiroyuki Futani, Takayuki Kawaguchi, Tatsuo Sawai, Toshiya Tachibana

**Affiliations:** Department of Orthopaedic Surgery, Hyogo Medical University, 1-1 Mukogawa Nishinomiya, Hyogo 663-8501, Japan

**Keywords:** osteochondroma, fracture, surgery, knee, displacement, athlete, pediatric

## Abstract

**Purpose:** The main purpose of this study was to compare the clinical outcome of surgery versus observation in young athletes with fractured osteochondromas in the knee. The secondary aim was to evaluate displacement versus non-displacement fractures with regards to functional recovery. **Methods:** A retrospective analysis was performed in young athletes with fractures of osteochondromas in the knee. In the surgery group, resection of the osteochondromas was performed due to pain persisting at 4 weeks after injury. In contrast, patients with pain diminishing within 4 weeks after injury were observed without surgery. Displacement was defined as a gap widening of ≥1 mm between fragments, or translation of >50% of the distal fragment in relation to the proximal fragment. The time to return to the original sport was compared between groups. **Results:** The study sample was composed of 21 patients with a mean age of 12 years (range 9–16 years). There were 14 patients in the surgery group and 7 patients in the observation group. There were 10 patients (71%) with displacement and 4 patients (29%) with non-displacement fractures in the surgery group. Surgery was required more frequently in displacement than in non-displacement fracture patients (*p* = 0.01). The mean time to return to the original sport was 2.1 ± 1.1 and 7.2 ± 4.1 weeks in the surgery and observation groups, respectively (*p* < 0.01). **Conclusions:** Surgical excision is preferable in a young athlete’s knee presenting with displacement of fractured osteochondromas due to disabling symptoms and in order to allow them to return faster to original sports activities.

## 1. Introduction

Osteochondromas are benign cartilage-forming lesions, which account for 10–15% of all bone tumors and apparently occur in 2% of the population overall. Approximately 60% of osteochondromas are found in patients younger than 20 years [1]. Most solitary and multiple osteochondromas are usually asymptomatic. However, they can be symptomatic as a result of complications, such as a fractured osteochondroma, mechanical joint problems, vascular or neurological compromise, and secondary malignant changes (usually chondrosarcoma). Fracture of the osteochondroma is a rare complication. The pedunculated shape of an osteochondroma presents a risk of fracture due to the relative weakening of its stalk [2,3]. The mechanism of fracture includes either a direct blow injury or an indirect injury caused by the impingement of muscle or tendon [4]. Fracture of the osteochondroma causes symptoms unless there is union [5] or spontaneous regression [6]. A non-united fractured osteochondroma can cause disabling symptoms, such as persistent pain and restriction of joint movement [7]. Because teenage athletes tend to be physically active, there is a greater likelihood of direct or indirect injury to the osteochondroma, giving rise to fracture [4]. Therefore, it is important to establish the optimal treatment for this condition in young athletes.

With regard to management for osteochondromas, symptomatic lesions or lesions of malignant transformation require surgical resection, while asymptomatic lesions are observed without surgery [8]. Non-surgical treatment with observation could be a good option, since the majority of fractured osteochondromas eventually unite [5,9,10,11]. However, proper treatment of fractured osteochondromas is still controversial [10,11,12]. Importantly, there have been no previous papers addressing the relationship between the type of fractured osteochondroma and treatment options.

It is hypothesized that surgery is required in patients with persistent pain due to the displacement of fractured osteochondromas in order to obtain faster functional recovery. The main purpose of this study was to compare a surgical versus observational strategy in young athletes with fractured osteochondromas in the knee with regards to their clinical outcome. As a secondary aim, we evaluated the subgroups of displacement compared with non-displacement fractures with regards to time to return to original sports activities.

## 2. Materials and Methods

This is a retrospective comparative study, which was conducted at a single university hospital in Japan. Chart reviews were used for the retrieval of data on individual patients. This study was approved by our ethics institutional review board (No. 4132), and written informed consent was obtained from all patients. The predominant location of the fractured osteochondroma was the knee region (89%); proximal tibia, 14 (52%) and distal femur, 10 (37%), followed by proximal humerus, 2 (7%), and radius, 1 (4%).

### 2.1. Study Design and Sample Description

Between 2000 and 2020, 345 patients with osteochondromas were treated in our institute. A total of 27 patients (7.8%) were diagnosed with sports-related fracture of an osteochondroma. In the remaining 318 patients, 75 (23.6%) needed surgical resection due to symptomatic lesions (73 patients) or lesions of malignant transformation (2 patients).

The inclusion criteria for this study were the presence of sports-related fracture of osteochondromas in the knee region, located in the distal femur or the proximal tibia. All patients were less than 18 years old, with a follow-up period of more than 2 years. Exclusion criteria were lack of complete data and loss to follow-up. Among the 27 patients with fractured osteochondromas, 6 patients were excluded. Of the excluded patients, 2 patients had osteochondromas in the proximal humerus, 1 patient in the distal radius, and 2 patients were more than 18 years of age (30 and 31 years old), and 1 patient was followed for less than 2 years. Therefore, 21 patients met the inclusion criteria.

For the purpose of this study, we divided the patients in 2 groups according to treatment strategy. In the surgery group, resection of the entire osteochondroma was performed for the patients who could not restart their sports activities due to pain at the fracture site with abnormal physical findings at 4 weeks after the injury. In contrast, the patients who could restart sports activities without pain within 4 weeks without abnormal physical findings after the injury were treated without surgery and were placed in the observation group. The patients were allowed to restart sports activities as tolerated. The senior author (H.F.) performed all surgeries and provided pre- and postoperative management for all patients. As subgroup analyses, the differences between displacement and non-displacement osteochondroma fractures were evaluated in the surgery and observation groups, respectively.

In the present study, we made the decision regarding either surgery or observation at 4 weeks after the injury. This decision was based upon an experimental model showing that bone union by enchondral ossification occurred at the fracture site over a period of up to 4 weeks [13].

All patients were examined within 2 days after their injuries. Treatment by nonsteroidal anti-inflammatory drugs (NSAIDs) started from the consultation day. NSAIDs were provided for between 2 and 5 days depending on the severity of pain. At 4 weeks after the injury, the decision regarding either surgery or observation was made according to the patients’ complaint in addition to the physical findings. The physical findings were based on inflammatory signs and tenderness at the fracture sites.

In the surgery group, complete tumor resection was performed by removing the osteochondroma at the normal bone base. Therefore, total resection of the osteochondroma could be achieved by removing not only the distal fragment, but also the proximal fragment. Special attention was paid with consequential removal of the cartilage cap and perichondrium in order to minimize the risk of local recurrence [8].

No immobilization devices, such as casts or braces, were utilized in any of the patient groups.

### 2.2. Clinical Analysis

The mechanism of fracture was divided into direct and indirect injury [6]. According to patient history, direct injury was defined as a fracture due to a blow injury, and indirect as a fracture without history of a blow injury. The athletes were divided into high-level of ≥7 and low-level of <7 according to the Tegner activity score. The Tegner activity score is a one-item score which grades activity based on work and sports activities on a scale from 0 to 10. A score of 0 represents disability due to knee problems and 10 represents the activity of a national- or international-level soccer player [14]. A Tegner activity score of 7 to 10 is a high baseline physical activity level [15].

The time to return to the original sport was defined as the time between the surgery and the return to original sports activities at one’s preinjury level of performance for the patients with surgery. For the patients without surgery, it was defined as the time between the injury and the return to original sports activities at one’s preinjury level of performance.

### 2.3. Radiographical Analysis

**Figure 1:**
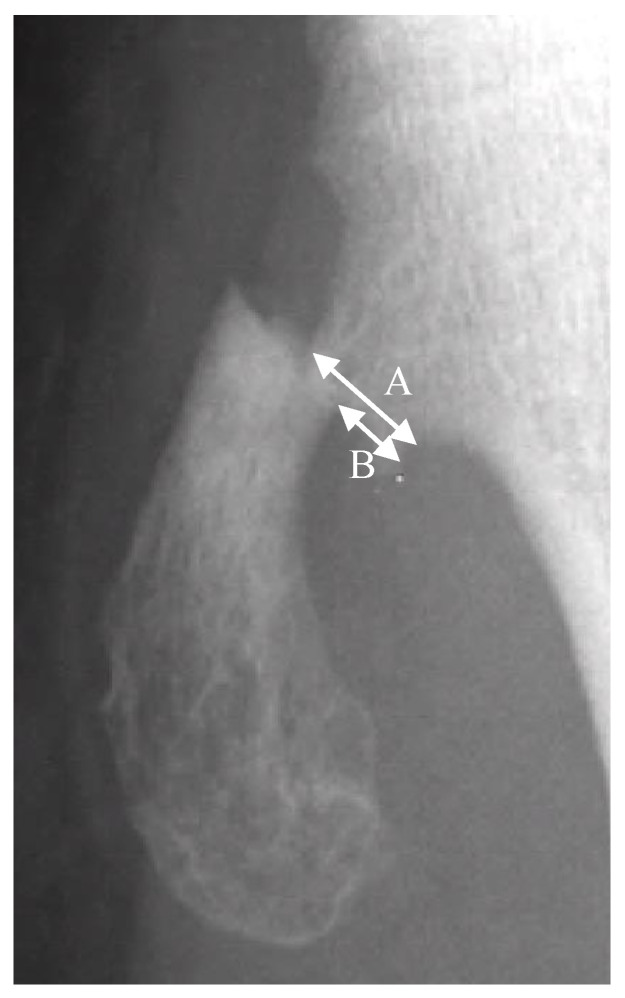
A is the width of the proximal fragment of the fractured osteochondroma. B is the displaced distance of the distal fragment in relation to the proximal fragment. Translation is defined by the equation of B/A × 100%.

**Figure 2:**
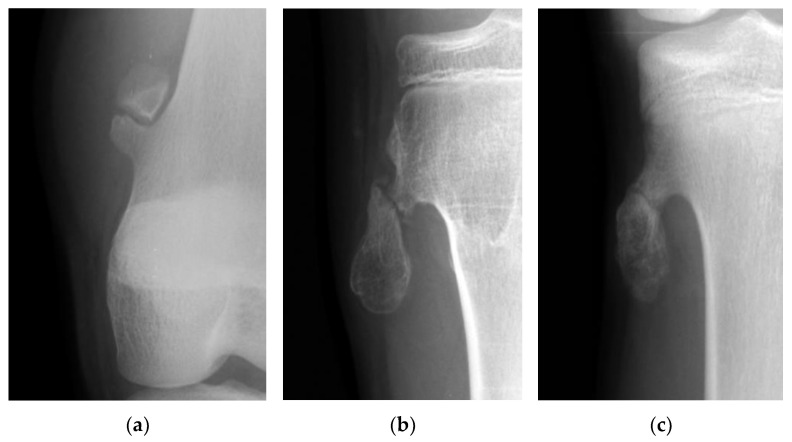
(**a**) The picture shows a gap widening of more than 1 mm between fragments of an osteochondroma in the femur. (**b**) The picture shows a more than 50% translation of a fractured osteochondroma in the tibia. (**c**) The picture shows a fractured osteochondroma in the tibia from the non-displacement group.

The fractures of the osteochondromas were divided into two types: displacement and non-displacement. In the present study, displacement was defined as a gap widening of more than 1 mm between fragments, or translation of more than 50% of the distal fragment in relation to the proximal fragment, either by anteroposterior, lateral, or oblique radiographic views of the knee joint (Figure 1). There were 12 patients in the displacement group and 11 patients in the non-displacement group (Figure 2).

Union of the fractured osteochondromas was defined as an ossified union between the distal and proximal fragments at the fracture site according to radiographic views of the knee joint.

All radiographic parameters were analyzed by 3 doctors (H.F., T.K., and T.S.) individually. One doctor was an orthopedic surgeon with 34 years of experience. The other two doctors were orthopedic surgeons with 7 years of experience. These observers were blinded to the outcome, and to each other’s results.

Patients were followed up by both clinical and radiographical examinations at the 1st week, 2nd week, 3rd week, and then at the 1st month, 2nd month, 3rd month, 6th month, 12th month, and annually thereafter.

### 2.4. Statistical Analysis

Data were presented as mean and standard deviation for continuous variables and frequency and percentage for categorical variables. Groups were compared using Student’s or Welch’s *T*-test for continuous variables, and a chi-squared test or Fisher’s exact test for categorical variables. All analyses were performed using the Statistical Package for the Social Sciences (SPSS, version 27.0, IBM Corp., Armonk, NY, USA). A *p* value of <0.05 was considered to be statistically significant.

## 3. Results

### 3.1. Patients’ Characteristics

Patient demographics and baseline clinical characteristics are shown in Table 1.

The mean age of the 21 included patients was 12 years old (range 9–16 years old). Regarding sports activities, soccer and tennis were the most frequent.

A solitary osteochondroma was diagnosed in 18 patients (86%) and multiple osteochondromas in 3 patients (14%). Fractures of the osteochondromas were found in the femur of 8 patients (38%) and in the tibia of 13 patients (62%). Indirect injury was found to be more frequent, present in 17 patients (81%), than direct blow injury, present in 4 patients (19%). The mean follow-up period was 48 months.

### 3.2. Clinical Outcome of Surgery and Observation Groups (Table 2)

There were 14 patients in the surgery group and 7 patients in the observation group.

Fractures of the osteochondromas were found in the femur and the tibia in 50% each of the patients in the surgery group. In the observation group, the fracture was found in the femur in 14% and in the tibia in 86%. Regarding the mechanism of fracture, direct blow injury was found in 21% and indirect in 79% in the surgery group. Direct blow injury was found in 14% and indirect in 86% in the observation group. These findings were not statistically significant. Furthermore, no statistically significant differences were found in age, sex, level of athletes, and follow-up period between the two groups.

Displacement fractures required surgery in 10 out of 11 patients (91%) at 4 weeks after injury. In contrast, only 4 out of 10 (40%) patients with non-displacement fractures required surgery (*p* = 0.01).

The mean time to return to the original sports activities was 2.1 ± 1.1 weeks (range 1–4 weeks) and 7.2 ± 4.1 weeks (range 3–15 weeks) in the surgery and observation groups, respectively (*p* < 0.001).

Neither postoperative complications nor local recurrence were found in the surgery group.

**Table 2:** Clinical Outcome of Surgery and Observation Groups.

Clinical Outcome	Surgery Group (n = 14)	Observation Group (n = 7)	*p* Value
Age, years	12.1 ± 1.6	12.9 ± 2.0	0.39
Sex, male/female	8/6	3/4	0.54
Tumor site, femur/tibia	7/7	1/6	0.11
Mechanism, direct/indirect	3/11	1/6	0.69
Level of athletes by Tegner activity score, ≥7/<7	6/8	3/4	0.54
Displacement/Non-displacement	10/4	1/6	0.01 *
Time to return to original sport, weeks	2.1 ± 1.1	7.2 ± 4.1	<0.01 *
Follow-up periods, months	45.0 ± 37.4	53.7 ± 27.9	0.59

* Statistically significant (*p* < 0.05).

### 3.3. Clinical Outcome of Surgery and Observation in Displacement Fractures (Table 3)

Surgery was required in 10 out of 11 patients (91%) with displacement fractures.

**Figure 3:**
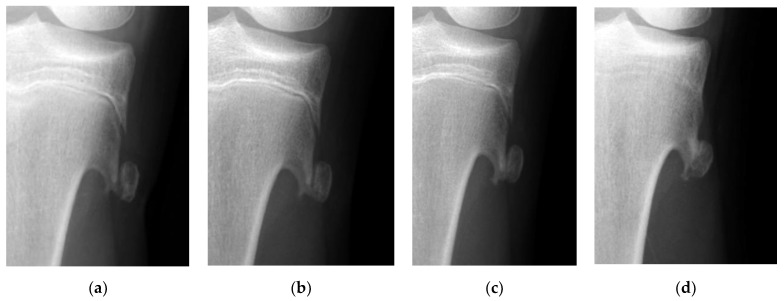
The radiographic views show sequential changes of the displacement of a fractured osteochondroma in the tibia. (**a**) Displacement of the fractured tibial osteochondroma is found in a 13-years-old girl who is a tennis player. (**b**) Union cannot be observed 4 weeks after the injury. (**c**) Partial union is achieved 1 year after the injury. (**d**) Complete union can be observed 5 years after the injury.

The mean time to return to the original sports activities was 2.3 weeks (range 1–4 weeks) in the surgery group. In contrast, the longest time required to return to original sports activities was 15 weeks in the one patient in the observation group. Surgery was not performed since pain subsided within 4 weeks after injury. However, this patient could not return to tennis due to the discomfort caused by a prolonged union; it took as long as 54 weeks to achieve partial union (Figure 3).

**Table 3:** Clinical Outcome of Surgery and Observation in Displacement fractures.

Clinical Outcome	Surgery Group (n = 10)	Observation Group (n = 1)
Age, years	12.2 ± 1.6	13
Sex, male/female	5/5	0/1
Tumor site, femur/tibia	4/6	0/1
Mechanism, direct/indirect	1/9	0/1
Level of athletes by Tegner activity score, ≥7/<7	4/6	0/1
Time to return to original sport, weeks	2.3 ± 1.2	15
Time to obtain bone union, weeks	NA	54
Follow-up periods, months	53.4 ± 40.7	66

### 3.4. Clinical Outcome of Surgery and Observation in Non-Displacement Fractures (Table 4)

There were four patients in the surgery group and six patients in the observation group.

The mean time to return to the original sports activities was 1.5 weeks (range 1–2 weeks) in the surgery group. In contrast, the mean time for the observation group was 6.0 weeks (range 3–8).

**Table 4:** Clinical Outcome of Surgery and Observation in Non-displacement fractures.

Clinical Outcome	Surgery Group (n = 4)	Observation Group (n = 6)	*p* Value
Age, years	12.0 ± 1.8	12.8 ± 2.2	0.55
Sex, male/female	3/1	3/3	0.57
Tumor site, femur/tibia	3/1	1/5	0.19
Mechanism, direct/indirect	2/2	1/5	0.50
Level of athletes by Tegner activity score, ≥7/<7	2/2	4/2	1.00
Time to return to original sport, weeks	1.5 ± 0.6	6.0 ± 2.4	<0.01 *
Time to obtain bone union, weeks	NA	5.3 ± 2.1	NA
Follow-up periods, months	27.2 ± 3.8	51.7 ± 30.0	0.10

* Statistically significant (*p* < 0.05).

### 3.5. Clinical Outcome of Displacement and Non-Displacement in Surgery Group (Table 5)

There were 10 out of 14 patients (71%) with displacement fractures and 4 out of 14 patients (29%) with non-displacement fractures in the surgery group.

Regarding the mechanism of fracture, direct blow injury was found in 10% and indirect injury in 90% of patients with displacement fractures. Direct blow injury was found in 50% and indirect injury in 50% in patients with non-displacement fractures. The incidence of indirect injury tended to be higher than that of direct blow injury in displacement fractures (*p* = 0.1).

The mean time to return to the original sports activities was 2.3 ± 1.2 weeks (range 1–4 weeks) and 1.5 ± 0.6 weeks (range 1–4 weeks) for patients with displacement and non-displacement fractures, respectively (*p* = 0.22).

**Table 5:** Clinical Outcome of Displacement and Non-displacement fractures in Surgery group.

Clinical Outcome	Displacement Type (n = 10)	Non-Displacement Type (n = 4)	*p* Value
Age, years	12.2 ± 1.6	12.0 ± 1.8	0.85
Sex, male/female	5/5	3/1	0.39
Tumor site, femur/tibia	4/6	3/1	0.24
Mechanism, direct/indirect	1/9	2/2	0.10
Level of athletes by Tegner activity score, ≥7/<7	4/6	2/2	0.77
Time to return to original sport, weeks	2.3 ± 1.2	1.5 ± 0.6	0.22
Follow-up periods, months	52.2 ± 42.7	27.3 ± 3.8	0.28

Statistically significant (*p* < 0.05).

### 3.6. Clinical Outcome of Displacement and Non-Displacement in Observation Group (Table 6)

There were one out of seven patients (14%) with displacement fractures and six out of seven patients (86%) with non-displacement fractures in the observation group.

The mean time to return to the original sports activities was 6.0 ± 2.4 weeks (range 3–8 weeks) in the patients with non-displacement fractures. Furthermore, the time to obtain union was 5.3 ± 2.1 weeks (range 4–8 weeks).

**Table 6:** Clinical Outcome of Displacement and Non-displacement in Observation group.

Clinical Outcome	Displacement Type (n = 1)	Non-Displacement Type (n = 6)
Age, years	13	12.8 ± 2.2
Sex, male/female	0/1	3/3
Tumor site, femur/tibia	0/1	1/5
Mechanism, direct/indirect	0/1	1/5
Level of athletes by Tegner activity score, ≥7/<7	0/1	4/2
Time to return to original sport, weeks	15	6.0 ± 2.4
Time to obtain bone union, weeks	54	5.3 ± 2.1
Follow-up periods, months	66	51.7 ± 30.0

## 4. Discussion

To our knowledge, this is the first study in a sizeable number of young athletes with fractured osteochondromas in the knee region showing that surgery was required more frequently in the patients with displacement fractures than in those with non-displacement fractures due to disabling symptoms. The mean time to return to original sports activities was shorter in patients who required surgery (range 1–4 weeks) than in the ones who were observed without intervention (range 3–15 weeks). The presented results are expected to provide valuable information for the treatment of fractured osteochondromas. 

The incidence of fractured osteochondroma at any site has been reported between 5% to 6% [4,16]. The predilection site of this condition is predominantly the knee, in 50% to 86% of reported cases [4,16]. In the present study, the incidence of fractured osteochondroma was 8% (27 out of 345 patients) and the predominant location was the knee, in 89% (24 out of 27 patients), in accordance with the previous studies.

With regard to the mechanism of fractured osteochondromas, the Carpintero study [4] showed that indirect injury was present in four out of six patients (67%). This study was a case series (Level of evidence, 4). In our study, we found 17 out of 21 patients (81%) with indirect injury. These findings indicate that fractured osteochondromas could be caused by an impingement of muscle or tendon more often than direct injury during sports activities. In contrast, a direct blow injury was found in only four patients (19%). Direct blow injuries are more frequently caused by contact or jumping sports among pediatric patients [17]. In the present study, the five major sports activities causing fractured osteochondromas were soccer, tennis, track and field, basketball, and gymnastics. Among the 5 major sports, non-contact sports were performed by 13 patients (62%), whereas contact sports were performed by 8 patients (38%). Thus, fractured osteochondromas could occur more frequently in non-contact sports due to indirect injury. 

Osteochondromas represent pedunculated or sessile bony prominences in continuity with the marrow and cortex of the host bone [2,18]. The majority of osteochondromas occur as solitary lesions, whereas approximately 15% of osteochondromas occur in the setting of multiple osteochondromas or hereditary multiple exostoses, an autosomal dominant disorder characterized by multiple osteochondromas [1]. The osteochondroma is an abnormal growth from the normal bone, thought to be a developmental lesion, unlike a true neoplasm [3]. The osteochondroma is histologically composed of a cartilage cap in the top of the lesion, with enchondral ossification occurring in the base [2]. Fractured osteochondromas have the potential to unite by enchondral ossification, similar to regular bone [10]. Union of the regular bone can be achieved by primary or secondary fracture healing. Primary fracture healing requires correct anatomical reduction with no gap at the fracture site. Secondary healing is the most common form of bone healing and usually consists of only endochondral ossification [13]. When displacement occurs at the fracture site of the osteochondroma, fracture healing is difficult to achieve by either primary or secondary processes. Abnormal movement at the non-healed fracture site creates disabling symptoms. Consequently, surgery should be performed in patients with displacement. In the present study, surgery was required in 11 out of 12 patients with displacement due to persistent pain.

The optimal time for decision-making regarding either surgery or observation has not been established. Prakash et al. [19] reported that a 21-year-old patient with displacement of a fractured osteochondroma in the femur required surgery due to persistent pain with no radiological signs of union 6 weeks after the injury. Errani et al. [10] reported that an 8-year-old patient with non-displacement of a fractured osteochondroma in the femur was observed without surgery due to the disappearance of pain with radiological signs of union 4 weeks after the injury. These authors suggested that the time for decision-making should be between 4 and 8 weeks; however, their experience is based on only one case report each. In the study by Davids et al. [5], three patients with displacement of fractured tibial osteochondromas were treated. Surgery was performed in one patient due to persistent pain caused by non-union for as long as 1 year after the injury. Union of fractured osteochondromas was found at an average of 5 weeks by radiography in patients with non-displacement. With these regards, four patients with non-displacement fractures of osteochondromas were advised to undergo surgery at 4 weeks after the injury. However, these patients might have had pain relief if the observation period had been extended.

Surgery can be performed by resection of the distal fragment only, or of the entire tumor. Barton et al. [9] reported resection of the distal fragment with a small incision. However, it would probably grow again from its base, so it is better to excise the entire tumor completely. In the present study, local recurrence could did not occur, as we performed entire tumor resection at the first surgery.

Previously, fractured osteochondroma in young athletes’ knees has only been addressed in one previous paper. Carpintero et al. [4] included six patients with fractured osteochondromas in the knee. The mean age of those patients was 17 years old (range 15–23). Surgery was performed in four patients, and the time to return to sports activities was within an average of 4 weeks. However, the other two patients did not have surgery and it took more than 8 weeks for them to return to their original sports activities. Accordingly, these authors concluded that surgery might be preferable for an athlete with a fractured osteochondroma, to obtain a shorter recovery period. In the present study, we found a significantly shorter time to return to original sports activities in young patients undergoing surgery in comparison to those only under observation.

We included 21 young patients with fractured osteochondromas in our study, which currently is the largest number presented in the literature. Even so, one limitation is that our study might have been underpowered, thus decreasing the likelihood to detect any significant differences in outcome measures between the groups. Another limitation is that this study population consisted of patients with mixed clinical characteristics, including location of the fracture and sports activities. Finally, this study was single-center and was not randomized. In the future, a prospective, randomized, multi-center clinical trial should be conducted to confirm our findings. 

## 5. Conclusions

Surgical excision is preferable in a young athlete’s knee presenting with displacement of fractured osteochondromas due to disabling symptoms and in order to allow them to return faster to their original sports activities. We believe that this paper provides useful information on the treatment strategy of fractured osteochondroma in a young athlete’s knee.

## Figures and Tables

**Table 1:** Patient Demographics and Baseline Clinical Characteristics.

Characteristic	Value
Age, years	12.4 ± 1.7
Sex, male/female	11/10
Side, right/left	17/6
Sports activity	
Soccer	5
Tennis	5
Track and field	4
Basketball	3
Gymnastics	2
Hip hop dance	1
Swimming	1
Tegner activity score	6.4 ± 1.3 (range 3–9)
Solitary/multiple	18/3
Site of fracture, femur/tibia	8/13
Mechanism, direct/indirect	4/17
Displacement/non-displacement	11/10
Surgery/observation	14/7
Follow-up duration, months	47.9 ± 31.0 (range 24–166)

## Data Availability

The data associated with the paper are not publicly available, but are available from the corresponding author upon reasonable request.
